# More allogrooming is followed by higher physiological stress in wild female baboons

**DOI:** 10.1098/rsbl.2024.0163

**Published:** 2024-08-07

**Authors:** Charlotte Christensen, Anna M. Bracken, M. Justin O'Riain, Michael Heistermann, Andrew J. King, Ines Fürtbauer

**Affiliations:** ^1^ Department of Biosciences, Faculty of Science and Engineering, Swansea University, Swansea SA2 8PP, UK; ^2^ Department of Evolutionary Biology and Environmental Science, University of Zurich, Zurich 8057, Switzerland; ^3^ School of Biodiversity, One Health and Veterinary Medicine, University of Glasgow, Glasgow G12 8QQ, UK; ^4^ Department of Biological Science, Institute for Communities and Wildlife in Africa, University of Cape Town, Rondebosch 7701, South Africa; ^5^ Endocrinology Laboratory, German Primate Centre, Göttingen 37077, Germany

**Keywords:** accelerometry, grooming, HPA-axis activity, physiological stress, primates, sociality–health–fitness

## Abstract

Social bonds increase fitness in a range of mammals. One pathway by which social bonds may increase fitness is by reducing the exposure to physiological stress, i.e. glucocorticoid (GC) hormones, that can be detrimental to health and survival. This is achieved through downregulating hypothalamic–pituitary–adrenal (HPA)-axis activity. Indeed, long-term measures of social (grooming) bonds are often negatively correlated with HPA-axis activity. However, the proximate role of physical touch through allogrooming remains an open question in the sociality–health–fitness debate. Demonstrating the potential anxiolytic benefits of grooming in the wild is hindered by methodological limitations. Here, we match accelerometer-identified grooming in wild female chacma baboons (*Papio ursinus*) to non-invasive faecal GC metabolite concentrations (fGCs). Consistent with previous work, we found a negative (but statistically non-significant) overall relationship between individual averaged fGCs and grooming rates. However, when time-matching grooming to fGCs, we found that both more giving and receiving grooming were followed by higher fGCs. This upregulation of HPA-axis activity suggests that maintaining social bonds (and its ultimate fitness benefits) may come at a shorter-term physiological cost. This finding sheds new light on a ubiquitous social behaviour typically considered ‘relaxing’ and suggests that sociopositive contact can trigger physiological stress.

## 1. Introduction

Social bonds impact fitness [1] and long-term studies on social mammals of many taxa have demonstrated increased survival and reproductive success for individuals with strong social bonds [2–8]. Social bonds can facilitate access to fitness-relevant resources, including thermoregulatory benefits [9,10], food through increased tolerance [11–13] and protection against predators [14,15] or aggressive conspecifics [16,17]. Additionally, social bonds may confer fitness benefits through modulating hypothalamic–pituitary–adrenal (HPA)-axis activity [18]. HPA-axis activity involves the release of glucocorticoid (GC) hormones [19], which serve important functions in maintaining homeostasis during environmental and life-history challenges (e.g. maturation and reproduction [19,20]). However, these can be detrimental to health and survival upon high cumulative exposure [21–23]. In mammals, the presence of social bonds is often negatively associated with GC concentrations, both in ‘stressful’ contexts (‘social buffering’; [24–26]) and in everyday situations (‘main effect’; [27,28]), suggesting a shielding role of social bonds against these detrimental effects.

In primates, social bonds are maintained through allogrooming (the social grooming of conspecifics; hereafter: ‘grooming’) [29,30] and have ultimate fitness benefits (see above). However, it is not clear whether grooming serves a ‘means to an end’ (i.e. servicing social bonds), ultimately leading to lower HPA-axis activity overall, or whether it has direct (short-term) attenuating effects on HPA-axis activity. The former would suggest that the physiological benefits are an indirect consequence of grooming, where forged social bonds provide increased access to resources (see above) or alter individuals’ perception or experience of stressors (*sensu* ‘social buffering’; [18,24]). The latter would suggest that grooming itself directly reduces HPA-axis activity, potentially through a neurochemical pathway mediated by social touch [31–33]. To address this uncertainty in the sociality–health–fitness debate, it is necessary to distinguish between these potential short- and long-term benefits of grooming [34,35].

The short-term beneficial effects of receiving grooming often described as ‘hedonic’ or ‘tension-reducing’ seem intuitive [36]. In primates, recipients of grooming show decreased heart rate [37,38] and lower behavioural indices of stress [39–42]. Similarly, horses and cows show reduced heart rates when being groomed [43,44]. Oxytocin and endorphins are two key neuropeptides that are released when being groomed and both have ‘rewarding’ effects (reviewed in [36,45]). Oxytocin down-regulates HPA-axis activity [46] and thus may be important in down-regulating HPA-axis activity in grooming contexts [47,48]. Studies on humans [49,50], dogs [51] and fish [52] have shown that physical contact can reduce GC levels; however, to our knowledge, there are no studies on wild animals that directly link receiving grooming to HPA-axis activity. In contrast to receiving grooming, giving grooming can be seen as paying a cost (e.g. the traded commodity in biological markets: [34,53]). However, giving grooming is also followed by lower behavioural indices of stress in primates [41,42,54] and birds [55], suggesting that it can be ‘self-rewarding’ [56]. In humans, being prosocial (e.g. helping others) is linked to stress-buffering effects [57,58].

To date, most studies on the physiological correlates of grooming in the wild link average faecal glucocorticoid metabolite concentrations (fGCs) to average grooming rates over a fixed period ranging from one to three months [59–64]. These studies have been instrumental in illustrating the presence (or absence) of a long-term relationship between grooming and HPA-axis activity; however, testing for short-term effects has remained challenging. To time-match grooming to subsequent GC responses, an observer (traditionally) would have to continuously record all grooming interactions of an individual until collection of an opportunistic faecal (or urine) sample that falls within the appropriate time window (accounting for a hormone excretion lag of several hours or days depending on sample type [65]). While such protocols are possible, they are time-consuming (see [27,66]), and it is here that accelerometers—which allow for the continuous quantification of grooming [67]—can vastly increase the opportunities to investigate direct effects of grooming on HPA-axis activity.

Here, we investigate short- and long-term relationships between grooming and HPA-axis activity in wild female chacma baboons using non-invasive hormone analysis [68] and tri-axial accelerometry for continuous quantification of grooming activity [67]. We focus on females as they are philopatric and allocate considerable time to grooming to maintain close social bonds within the troop [69]. First, we test for an expected overall negative correlation between average grooming rates and average fGCs across the study period ([63,64,70] but see [59,60]). Second, we test for short-term effects of grooming on HPA-axis activity through time-matching grooming and fGCs. Based on the available literature on behavioural indices of reduced stress during and immediately after receiving [37–40] and giving [41,54] grooming, we expect that more time spent grooming should be followed by lower fGCs.

## 2. Methods

### (a) Study site and subjects

**Figure 1.**
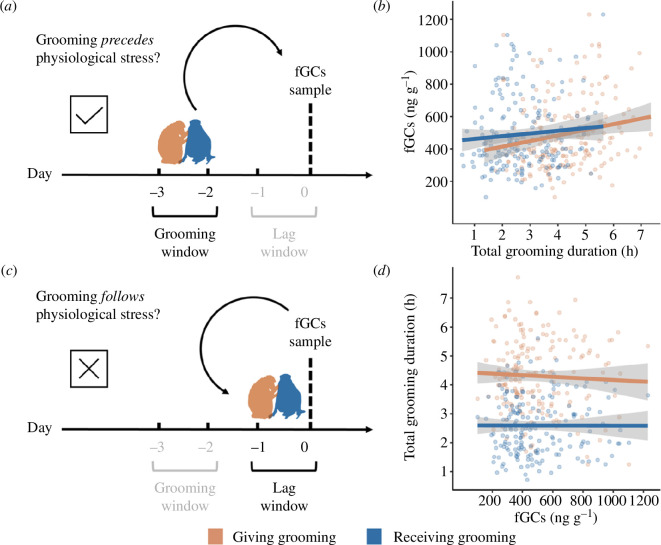
Relationship between grooming and HPA-axis activity. (*a*) To determine whether grooming predicted physiological stress, we matched a 2-day ‘grooming window’ (total grooming recorded on day −3 and −2) preceding a 2-day ‘lag window’ (accounting for the delay in hormone excretion in faeces) to fGCs. (*b*) Effect of giving (orange) and receiving (blue) grooming on fGCs (ng g^−1^) (LMM1). (*c*) To determine whether ‘stress status’ affected grooming during the ‘lag window’, we matched fGCs to total grooming recorded on day −1 and 0. (*d*) Effect of fGCs (ng g^−1^) on giving (orange; LMM2) and receiving grooming (blue; LMM3) during the ‘lag window’. In (*a*) and (*c*), the dotted line denotes the day the faecal sample was collected (day 0) from which the fGCs measurement was derived. In (*b*) and (*d*), the dots are the raw data points, and the solid line is the linear regression line with 95% confidence intervals in grey.

We studied a troop of wild chacma baboons consisting of approximately 50 individuals (*n* = 21 adults) in Da Gama Park, Western Cape, South Africa (−34.15562°N, 18.39858°E) between July and November 2018 (see [71,72] for study details). We use data for *n* = 10 adult females for which we have continuous accelerometer-identified grooming data [67] that can be time-matched to fGCs (figure 1) [68].

### (b) Grooming data from accelerometers

Giving and receiving grooming durations were obtained from collar-fitted tri-axial accelerometers, using random forest models trained on video-labelled accelerometer data (for details see [67]). Owing to the confusion between ‘receiving grooming’ and ‘resting’ (i.e. sleeping) by the random forest model at night, we only use daytime grooming data for all analyses.

### (c) Hormone data

Details on hormone sample collection, processing and analysis have been previously described in detail [68]. In brief, faecal extracts were analysed for immunoreactive 11β-hydroxyetiocholanolone [73], a major metabolite of cortisol, using a validated enzyme immunoassay previously used successfully to assess HPA-axis activity in the study species/population [68]. Intra- and inter-assay coefficients of variation were <8% and<12%, respectively. fGCs are expressed in nanograms per gram of dry weight.

### (d) Statistical analysis

All analyses were run in R Studio (R v. 4.1.1; studio v. 1.4.1717). Linear mixed models (LMMs) were run using the package ‘lmerTest’ [74]. Response and predictor variables were transformed to meet normal distribution where necessary and continuous predictor variables were z-transformed to make estimates comparable. Normality assumptions were visually confirmed by plotting residuals in Q–Q plots (function ‘qqnorm’). We tested for collinearity between predictor variables (package ‘car’ [75]). All predictors included in the models had variation inflation factors (VIFs) below 1.1 (VIF < 3 is considered a stringent cut-off to rule out collinearity: [76]).

### (e) Long-term relationship between grooming and faecal glucocorticoids

To test for a relationship between average accelerometer-identified grooming rates and average fGCs, we used a Spearman’s correlation (*n* = 7 females). As gestation is associated with increased fGCs [68,77,78], and reproductive state cannot be controlled for in simple correlations, we excluded *n* = 3 pregnant females from this analysis. Grooming rates were calculated by dividing the total number of seconds receiving or giving grooming during the day divided by total daytime across all full days (time between sunrise and sunset obtained from www.timeanddate.com; mean ± s.d. = 55 ± 18 accelerometer days and mean ± s.d. = 23 ± 6 fGCs samples per individual).

### (f) Short-term effect of grooming on faecal glucocorticoids

To test whether more grooming resulted in lower fGCs we had to (i) account for the time lag between grooming and the associated GC metabolites that are measured in excreted faeces (‘lag window’; figure 1) and (ii) establish a meaningful time window over which to calculate total grooming time (‘grooming window’; figure 1). For female baboons (*Papio cynocephalus cynocephalus*) infused with radio-labelled steroid hormones, peak excretion in faeces was 36.4 h after steroid infusion [79]. Accordingly, baboon studies have used 2-day lags between fGCs and the variable of interest [80,81]. This also is in line with the biological validation in our study troop, where fGCs were significantly higher 2 days after a capture event during which the accelerometer collars were deployed (see [68] for further details). We therefore use a 2-day lag window to match fGCs to grooming (figure 1). For the ‘grooming window’, we had to strike a balance between including enough grooming data (fGCs provide a cumulative measure of HPA-axis activity [65,68] and grooming is not an ‘acute’ event) but not too much grooming data (because we aimed to capture the physiological response that may be resulting from these grooming interactions). Following the only other study that time-matched behavioural data to fGCs [66], we used a window that matches the time-lag for GC excretion into faeces (i.e. 2 days; see above). Thus, we test the effect of grooming recorded over 2 days (‘grooming window’ on days −2 and −3; figure 1), preceding a 2-day lag (‘lag window’ day −1 and day 0; figure 1) from the day of faecal sample collection (day 0).

A total of *n* = 185 fGCs could be matched to accelerometer-derived grooming data. To test for the short-term effect of grooming on fGCs (LMM1), we fitted total duration of giving and receiving grooming across days −2 and −3 as a fixed effects (square root and z-transformed) with fGCs (log-transformed) as the response variable. We controlled for reproductive state (categorical: pregnant/non-pregnant), day length (continuous: time between sunrise and sunset, averaged between day −3 and −2) and urine contamination (categorical: yes/no), based on previous work [68] and included ‘date’ and ‘baboon ID’ as random intercepts to control for individual differences and uneven sampling across days [68].

### (g) Effect of physiological stress on grooming activity

A positive relationship between grooming and HPA-axis activity (LMM1) could be interpreted as an underlying stressor causing a concurrent behavioural and physiological response [82]. If this was the case, we would expect periods of increased physiological stress to be marked by more grooming generally, and therefore more grooming should follow, not just precede (LMM1), high HPA-axis activity. We tested whether ‘stress status’ (i.e. fGCs) predicted giving (LMM2) and receiving (LMM3) grooming during the 2-day ‘lag window’. The absence of a relationship would suggest that HPA-axis activity did not affect grooming, as the ‘lag window’ should capture grooming that followed the measured HPA-axis activity. We control for predictors of grooming time in our study troop [83], i.e. day length (time between sunrise and sunset, averaged between day −1 and 0) and again included ‘date’ and ‘baboon ID’ as random intercepts.

## 3. Results

We found a negative (but non-significant) relationship between individual mean grooming duration and individual mean fGCs (Spearman’s rho = −0.75, *p* = 0.066, *n* = 7; electronic supplementary material, figure S1). In the short term, both receiving and giving grooming during the ‘grooming window’ (days −3 and −2) were significantly positively associated with fGCs (figure 1*b*; table 1; LMM1). We found no effect of ‘stress status’ (fGCs) on giving (LMM2: estimate ± s.e. = −0.011 ± 0.015, *T* = −0.765, *p* = 0.451) or receiving grooming (LMM3: estimate ± s.e. = 0.026 ± 0.018, *T* = 1.403, *p* = 0.162) (figure 1*d*) during the ‘lag window’ (days −1 and 0).

**Table 1.** Effect of grooming on fGCs (LMM1, *n* = 10 females, *n* = 185 samples, *n* = 50 unique dates), controlling for day length, reproductive state and urine contamination, respectively. Significant effects are in bold.

predictor variable	estimate	s.e.	*T*	*p*
*fGCs* (LMM1)				
giving grooming (h)	0.092	0.039	2.362	**0.020**
receiving grooming (h)	0.096	0.041	2.371	**0.019**
day length (h)	−0.119	0.032	−3.763	**<0.001**
pregnant (Y)	0.335	0.112	2.994	**0.003**
urine contamination (Y)	0.060	0.079	0.758	0.450

## 4. Discussion

The present study addresses a crucial gap in the sociality–health–fitness debate [84] by testing whether grooming modulates HPA-axis activity in the short term. In line with previous work [63,70], we show a negative (but non-significant) relationship between average rates of grooming and average fGCs. Conversely, when time-matching grooming to fGCs, we found that more time giving and receiving grooming was associated with higher fGCs, contrary to our expectations (figure 1*b*). The positive time-matched relationship between grooming and HPA-axis activity could reflect a concurrent behavioural and physiological response to a stressor [82]. For instance, female baboons show increased fGCs and expand their grooming network and activity following the loss of a relative [64]. However, in our study, increased HPA-axis activity was not followed by more grooming (figure 1*d*), ruling out this explanation and suggesting a short-term cost of grooming.

Several papers have called for a re-examination of the costs and benefits of grooming [34,84,85], particularly giving grooming [63,86]. Giving grooming can be costly, as it can reduce vigilance [87–89] or carry energetic (picking through fur is laborious and may require cognitive resources [34,90]) or opportunity costs (grooming inevitably detracts time from other activities [91,92]). Moreover, grooming could lead to interference of others (e.g. competition for grooming partners [93,94]) and the groomer may be exposed to increased aggression by the recipient of the grooming interaction [95,96], who could be frustrated by the termination of the grooming bout ([40] but see [54,97]). For example, low-ranking mandrills (*Mandrillus sphinx*), preferentially groom ‘safe areas’ (e.g. rump and back), which allow time to flee in case of aggression by the recipient [98] and in Barbary macaques (*Macaca sylvanus*), self-scratching increases after giving grooming, potentially indicating a state of increased anxiety [86,99]. These behavioural studies support the interpretation that increased GC levels after giving grooming may be driven by psychosocial stress and emphasize that, at least in hierarchical societies, the consequences of grooming can be unpredictable [96]. It is surprising that receiving grooming was also associated with higher fGCs considering that, unlike giving grooming, most studies are equivocal about the relaxing effects of being groomed [37,38,41]. Nevertheless, it may carry similar costs to giving grooming in terms of opportunity costs and potential aggression or interferences from nearby group members. Moreover, in our study, seven out of 10 collared females had dependent infants of varying ages, which can affect grooming relationships [100,101]. Baboon mothers often receive unsolicited attention as infants attract other females [102,103], who groom the mothers to handle their infants [101]. Lactating females may thus have less control over who grooms them, potentially increasing HPA-axis activity [60].

To our knowledge, physiological or behavioural indicators of heightened stress during or directly following grooming (or preening) have not been recorded outside of primates, but a similar ‘appeasing’ function is often ascribed (e.g. Norway rats *Rattus norvegicus* [104], common guillemots (*Uria aalge*) [105] and meerkats *Suricata suricatta* [106]), suggesting these interactions can involve a potential aggressor and could therefore be associated with psychosocial stress. Further studies looking at short-term physiological and behavioural consequences of grooming (or preening) in different taxa are needed to confirm this. Moreover, to disentangle the different sources of costs linked to both giving and receiving grooming, future work combining GC, oxytocin and other relevant physiological measurements could help clarify whether the cost is energetic, psychosocial or both. Our finding that both giving and receiving grooming were associated with increased fGCs suggests a role of psychosocial stress, because only giving grooming is an active, potentially energetically costly (see e.g. [92]) behaviour.

The long-term benefits of increased social tolerance or access to resources through grooming [107,108] may explain why long-term rates of grooming are associated with reduced HPA-axis activity [63,64,70]. The negative relationship between individual average grooming rates and fGCs found here was not statistically significant, which may be owing to a small sample size (*n* = 7 baboons) or owing to other (related) grooming metrics (e.g. grooming network stability [60], balance [109], within-bout reciprocity [110], clique size [63,111], centrality [112] or partner [27,61]) being more important for long-term HPA-axis modulation. Regardless, these contrasting short- and long-term patterns in HPA-axis activity in response to grooming can be understood through the lens of comparing the ‘act of grooming’ to its long-term benefits. Grooming may be far from ‘relaxing’ if it is used as a mitigation strategy against exposure to imminent aggression. For example, in Barbary macaques, victims of aggression groom the aggressor to reduce the likelihood of renewed attacks [113] and in vervet monkeys, time spent giving grooming is positively correlated with the duration of tolerance around food by a higher-ranking grooming recipient [114]. A physiological cost of grooming also is in line with the ‘social bond testing’ hypothesis [115] which posits that bonds are tested by engaging in behaviours that are inherently costly or involve risk. For example, presenting vulnerable genital regions to a conspecific during ritualized greetings (spotted hyenas *Crocuta Crocuta* [116], olive baboons *Papio anubis* [117]) or a mother allowing ‘infant handling’ by other females (white-faced capuchins *Cebus capucinus* [118]) involves obvious risks and has therefore been argued to be a good indicator of bond strength. Although grooming is considered sociopositive, the associated risks (see examples above) may make engaging in grooming a good test of bonds as well. Ultimately, the grooming-incurred tolerance or social bond support may decrease exposure to future stressors, resulting in lowered HPA-axis activity in the long-term, but be physiologically costly in the short term. Note that similar paradoxical relationships between physiological stress and fitness have been also been hypothesized in inter-specific ‘grooming’ interactions. For instance, cleaning gobies (*Elacatinus evelynae*) show increased cortisol concentrations in the presence of predatory client fish, but by tending to them first and for extended periods, they may reduce their risk of being preyed upon [119].

In conclusion, we show that, in female chacma baboons, grooming has direct short-term physiological costs, with more grooming being followed by higher HPA-axis activity. This finding suggests that the long-term negative relationship between physiological stress and grooming ([63,70]; present study) is unlikely subserved by grooming itself (i.e. touch-mediated reduction in HPA-axis activity; [31–33]) but, instead, is tied to the incurred long-term benefits of grooming (e.g. access to fitness-relevant resources and services; [9–17]). This unexpected result has important implications for the wider sociality–health–fitness nexus [84] because it suggests that social bonds that ultimately affect survival [1] are maintained by a process (grooming) which—in the short term—can be physiologically costly. High-resolution grooming data from collars could be leveraged to study how this short-term hormone–behaviour relationship might change across environmental and social contexts and, if deployed in other social animals, confirm how widespread this phenomenon is.

## Data Availability

The ESM contains three files: (1) R script (fGCs_groom_publication_20240523.R) which calls on the two data frames and can be used to run the analysis for the short-term links between fGCs and grooming and long-term correlation between grooming and fGCs. The script contains the packages and explanation of each variable. (2) Grooming_fGCs_publication.rds: data frame with the samples associated with the grooming data and other variables controlled for in the analysis (e.g. reproductive state, environmental conditions) for n = 10 female baboons. (3) Grooming_fGCs_longterm_publication.rds: data frame with the rate of grooming and the average fGCs for n = 7 female baboons. Supplementary material available online [120].
